# Supplementary material for: Effects of social determinants of health on obesity among urban women of reproductive age
Source: PLOS Glob Public Health. 2023 Jan 24;3(1):e0001442. doi: 10.1371/journal.pgph.0001442 (PMC10022252; doi:10.1371/journal.pgph.0001442)
Supplement: Attachment — Submitted filename: Response to reviewers.docx [file pgph.0001442.s001.docx]

Dear Editor,

We thank you and the reviewers for reviewing our manuscript. We have addressed the reviewers’ comments and the details are presented below:

Reviewer #1:

COMMENT:  Firstly, in abstract no need to put BMI definition in abstract

RESPONSE: This has now been removed

COMMENT: In method: Why put low and middle income countries, you mean this is multi countries survey

RESPONSE: We thank the reviewer for this comment. For clarity, the sentence now reads “These are nationally representative data collected every five years in Ghana and other low and middle-income countries”

COMMENT: SDH is what?

RESPONSE: Social Determinants of Health

COMMENT: Introduction is too long, summerize

RESPONSE: We thank the reviewer for this comment. We tried to cut out some text from the introduction where possible. Nevertheless, we feel the introduction is not very long as it is only one and half pages on single spacing.

COMMENT: Study aim is not clear please clarify
Current analysis period (years)?

RESPONSE: We thank the reviewer for this comment. The year is now included.

The aim of the study is now stated as follows “The present study examined the effects of SDH on obesity among women of reproductive age in urban Ghana using existing data”

COMMENT: Only 6% agricultural so how come in conclusion work in agriculture is protective?

RESPONSE: We thank the reviewer for this comment. Our statistical modelling suggests that despite agriculture being 6%, it is protective. This probably because of the large sample size we have used, which makes 6% reasonable enough for the analysis.

COMMENT: How wealth index categorized?.

RESPONSE: We thank the reviewer for this comment. Although the wealth index is originally classified into five categories (poorest, poor, middle, rich & richest), we decided to recode it into three categories (poor, middle & rich) to preserve sample size for the analysis.

COMMENT: Table 3 R2 of the model?

RESPONSE: We thank the reviewer for this comment. We did not report R^2^ in table 3 because we felt in logistic regression modelling, reporting R^2^ is not necessary. Therefore, nothing is lost by excluding it in the tables.

COMMENT: Model 1 & Model 2 method of logestic regression?

RESPONSE: Yes, we employed logistic regression in our analysis.

COMMENT: Strength is 1.402 large enough?

RESPONSE: We thank the reviewer for this comment. In our view, we think 1,204 is large enough for our analysis.

Reviewer #2:

COMMENT: The author raises important issues that need to be explored the evidence by research. The evidence generates importance in providing an appropriate implementation in the reduction of obesity-related morbidity and mortality. However, two amendments are needed to improve the quality of the research.

RESPONSE: We thank the reviewer for this positive comment.

COMMENT: 1. Try to analyses Minimum dietary diversity for women instead of considering only fruit and vegetable consumption.

RESPONSE: We thank the reviewer for this comment. Unfortunately, the data did not capture variables that will make it possible for the computation of the minimum dietary diversity for women (MDD-W). Hence the decision to use fruit and vegetable consumption instead.

COMMENT: 2. Try to reconsider the relationship b/n higher education and obesity. Find deep review and justify how higher education is negative corelated obesity?

RESPONSE: We thank the reviewer for this comment. The reviewer’s concern is addressed in lines 115-123 of the introduction section and lines 269-280 in the discussion section.
